# Supplementary material for: Self-organizing actin patterns shape membrane architecture but not cell mechanics
Source: Nat Commun. 2017 Feb 13;8:14347. doi: 10.1038/ncomms14347 (PMC5316839; doi:10.1038/ncomms14347)
Supplement: Supplementary Information — Supplementary Notes, Supplementary Figures, Supplementary Table 1, Supplementary Discussion and Supplementary References [file ncomms14347-s1.pdf]

Supplementary Note 1

General remarks on the observations. We here briefly comment on the qualitative differences in the images of actin patterns using the different imaging techniques. In the case of fluorescence imaging such as STED microscopy and eTIRF-SIM, we cannot determine sizes (or lengths of the peripheral filaments) or fiber orientations of the patterns at the same spatial accuracy as provided by SEM, which would have allowed a continuous quantitative and statistically precise comparison of stars and asters. The reduced accuracy of the fluorescence microscopy recordings with respect to aster and star characterization mainly results from their still rather low spatial resolution along the axial direction (~100-300 nm compared to 20-50 nm for SEM), resulting in the additional appearance of out-of-axis features and making an automated allocation towards the star- and aster-like features inaccurate. Yet, the SEM recordings have to be performed on fixed cells, which impedes the direct study of dynamical events. Further, fluorescence microscopy allows the straightforward simultaneous specific study of different molecules (e.g. F-actin and actin-associating proteins with differently colored labels), since the experimental protocols for multi-label fluorescence microscopy recordings on living cells are far less challenging than for the fixed cells in SEM. In addition, the fluorescence recordings allow us to overview multiple cells and actin features in large field-of views.

Supplementary Note 2

Further details on the classification of self-organized actin patterns. As highlighted in the introduction and in Figure 1B, vortices are ring-like structures with high rotational symmetry, which distinguish themselves from the asterisk-like topology of stars and asters with their distinct nucleation centers and arm-like radially orientated F-actin strands. Asters and stars have so far not systematically been differentiated and their nomenclature has synonymously been interchanged without clear distinction [1,2]. On one hand, this inaccurate distinction results from the fact that both patterns share the same asterisk-like topology. On the other hand, the topology of star- or aster-like features (such as the core diameter or lengths of the F- actin arms, i.e. the total pattern-size) strictly depends on the biochemical and experimental settings used for observing the patterns. In in vitro experiments these are for example the concentration of free actin monomers available for actin polymerization. More recent work started to differentiate asters and stars displaying the latter as significantly larger than asters and further distinguished them by the characteristics of the arms radiating outwards from the center; these shall consist of multiple parallel bundled F-actin in stars rather than individual actin filaments in asters [3-5].

Supplementary Note 3

Transitions between actin stars and asters. We aimed to directly demonstrate that actin stars transform into asters. Yet, we have to note that our fluorescence observation techniques (eTIRF-SIM or STED microscopy) were not able to follow the full time course of these transformations over hours. Due to photobleaching of the fluorescent labels and potential phototoxic effects by the illumination, we were in our case limited to in maximum 300 frames long acquisitions at the different stages (Supplementary Figure 2). Yet, during these recordings we did not experience any changes in overall cell viability, and consequently the recordings at the different stages precluded that the pattern transformations were caused by phototoxic reactions. In addition to the so far not possible hour-long recording times, another reason why we were not able to directly show more occasions of the full transition between the three different patterns, is the relatively fast and spontaneous nature of the transitions; i.e. while the time duration until the different critical conditions are reached takes multiple hours the actual transition between the patterns has been shown to be fast, which are generally hard to capture and would require a microscopy tool allowing hour-long recordings with low phototoxicity and sub-100 nm spatial and sub-second temporal resolution; a technology enabling such recordings is currently not within our hands. Additionally, axial drifts currently preclude eTIRF-SIM measurements over multiple minutes to hours, which limited our observations times for instance in the case of the inhibition experiments.

Despite the aforementioned limitations, we were able to capture some transition events. eTIRF-SIM measurements of untreated live HeLa cells stably expressing fluorescent F-actin (Lifeact-citrine) were performed at intermediate to late incubation times (4-12 h). Supplementary Figure 4A shows a time-lapse of one actin star (blue arrow) transforming into two actin asters (red arrows) by dividing their actin cores and strands within a time period of about 40 s (Supplementary Movie 7). Quantifications of the corresponding line profiles through the cores of the actin features (right panel) display a single-peaked Gaussian intensity distribution (blue dashed line) prior star division and a double-peaked intensity distribution post star division (red dashed line), indicating that actin stars do naturally divide into actin asters.

Intriguingly at this one occasion, we also observed three actin asters to fuse into one actin star. Supplementary Figure 4B shows three spatially separated asters transforming into one actin star by fusing their actin cores and strands within a time period of about 120 s (Supplementary Movie 8). Quantifications of the corresponding line profiles (right panel) display a triple-peaked Gaussian intensity distribution (blue dashed line) prior aster fusion and a single- peaked intensity distribution post star division (red dashed line), indicating that actin asters can occasionally fuse into stars.

At other occasions, we were able to image how actin asters spontaneously transformed into randomly organized F-actin networks (Supplementary Figure 4C).

Our observations demonstrated that actin stars indeed transformed into asters by division of the star core and strands. In turn, asters may occasionally transform into stars by fusing their cores and strands. Additionally, asters may simply disintegrate into randomly organized F-actin networks. Quantifications of the relatively low number of directly observed transitions between asteriks-like structures (10 in total) revealed that the transitions from actin stars into asters were most frequent (9 out of 10), whereas aster fusions into actin stars were rather rare ($p < 0.01$ student t-test, 1 out of 10). This supported the notion of a natural sequence of transitions from

actin stars into asters and further on their disintegration, since the latter event was quite commonly observed.

The limited spatial accuracy of the fluorescence recordings precluded exact determination of the pattern density per cell at a given time point. In addition, not all cells featured clearly distinguishable star- or aster-like structures. The latter features are in parts difficult to identify within the crowded cortical actin mesh or simply not present. From our fluorescence microscopy experiments, we estimated a fraction of roughly 30% of all cells to show distinct patterns at the basal plane at a time. This heterogeneous behavior may result from varying adhesion characteristics across a single cell and between cells, especially from the cell-to-cell variation of the exact time point of attachment to the substrate. Nevertheless, the appearance and differentiation of stars and asters could still be well quantified from the fluorescence recordings, and, to support our previous observations, we quantified the likelihood of finding specific actin patterns at a given time point at the basal plane of cells (upper panel, Supplementary Figure 4D). After 2-3 h incubation we found that > 90% of all patterns were vortices (black) and only 5% stars (red) or asters (blue). After 4 h incubation actin stars were most prominent (> 90%) and only 5% vortices and asters were on average observed. The fraction of asters was the largest after 12 h incubation (80%), followed by 20% stars, and 0% vortices, suggesting a physiological sequence of transitions from actin vortices to stars and stars into asters. Further, we computed in these cells the total fraction of surface-area occupied by actin patterns by calculating the surface area occupied with certain actin patterns normalized to the total surface area at the basal plane of cells (lower panel, Supplementary Figure 4D). We found that vortices mostly occurred in larger ensembles occupying on average 70% of the surface-area after 2 h incubation if present at all (black). After 4h incubation where the large stars were the predominant pattern, these mostly occurred individually but were small compared to the total surface area of cells, and therefore occupied on average ~ 5% of the total surface-area (red). After 12h of incubation smaller asters were dominant, occurred mostly in larger ensembles and occupied on average ~ 50% of the total surface-area (blue).

Supplementary Note 4

Stars and asters are nucleated and maintained independent of myosin-II. We also determined the possible involvement of myosin-II motor proteins in the nucleation and maintenance of the star- and aster-like actin patterns. eTIRF-SIM measurements of untreated live HeLa cells stably expressing fluorescent F-actin (Lifeact-citrine) and myosin-II (halo-tagged myosin regulatory light chain 2 (MRLC2) labeled with the membrane permeable organic dye JF542) showed that myosin patches localized only to the F-actin strands of both stars and asters but hardly to their cores, with an active mobility along the filaments (Supplementary Figure 5A,B). In order to test whether myosin motors were essential for the maintenance of stars and asters, we pharmacologically blocked MRLC phosphorylation by the Rock/Rho-kinase inhibitor Y27632 (Supplementary Figure 5C,D). Yet, the actin cortex did not show any difference in the pattern organization on the total time scale of the eTIRF-SIM measurements, and both stars and asters maintained their characteristic structures. Consistent with the previous inhibition experiments, myosin-II was immobile in response to Y27632, highlighting the functionality of the drug. Surprisingly, quantifications of the localization of the myosin-II patches revealed that at control conditions and in the presence of Y27632 (or DMSO) effectively > 99% of all myosin were located at the star or aster strands but < 1% at the pattern cores (Supplementary Figure 5E,F). These observations suggested that myosin-II was not actively participating to pattern nucleation and maintenance.

Supplementary Note 5

Polarization states of actin stars and asters. As highlighted in the main text and Figure 5H-J, we performed FRAP (fluorescence recovery after photo-bleaching) experiments on actin filaments to report on their turnover dynamics. Specifically, we studied the fluorescence recovery of stably expressed fluorescently tagged F-actin (Lifeact-citrine) in live HeLa cells. In an ideal experiment, fluorescently tagged actin monomers would have been employed to report on the true actin turnover. Unfortunately, actin patterns are hardly visible and not reliably identifiable in the presence of fluorescently tagged free actin monomers in the cytoplasm of the cells, and therefore Lifeact was chosen as a marker for F-actin localization. Notably, the fluorescence recovery of Lifeact is a convolution of the assembly dynamics of actin and Lifeact itself; such as Lifeact's diffusion in the cytoplasm and its association/dissociation to F-actin. Typically, however association to F-actin by Lifeact is significantly faster than actin polymerization dynamics [6-8], and therefore do not affect recovery qualitatively and could be here employed to reveal F-actin polarity.

In the FRAP experiments, a small region of interest (ROI) is bleached by a short exposure to high-power laser light, and then fluorescence recovery is monitored over time. Such experiments reveal whether protein structures are active and undergo continuous remodeling or not. The results are generally quantified by monitoring the time scale of the fluorescence recovery and the fraction of protein that appeared immobile (on the timescale over which recovery was followed)[9]. In the current FRAP experiments, we specifically imaged a rectangular ROI and photo-bleached a smaller rectangular ROI (bROI) within this ROI. Fluorescence recovery was then monitored at a linear ROI (lnROI) parallel to actin fibers within the bROI over time (Figure 5H-J, left panels, and Materials and Methods).

Supplementary Note 6

Self-organizing actin patterns shape cortex-membrane interface. To gain further insights into the functional role of actin patterning, we investigated alterations in the lipid molecular order (or packing) und thus fluidity of the cell membrane in the presence and absence of the actin patterns. Specifically asters have theoretically been assumed to affect the architecture of the plasma membrane and thus the dynamics and functions of membrane-associated proteins and lipids [10]. Hence, we reasoned to measure the lipid order in the cellular membrane by using S-Laurdan2 (S-L2, derivative of 6-Dodecanoyl-2-Dimethylaminonaphthalene) [11]. S-L2 is a polarity-sensitive membrane-embedding dye, which changes its fluorescence brightness (with a maximum around 480 nm) depending on the polarity of its immediate membrane environment. More ordered (and thus less fluid) lipid membrane environments are characterized by a lower polarity due to the presence of less water molecules, and S-L2 in these environments exhibits an increased fluorescence brightness compared to less ordered (and thus more fluid) environments [12]. We labeled the plasma membrane and F-actin of the adherent HeLa cells with S-L2 and Lifeact-Citrine, respectively, and simultaneously imaged the spatial organization of both on the basal membrane using conventional confocal microscopy. The absorption and emission spectra and photostability of S-L2 were unfortunately not optimal for its use on the advanced STED or eTIRF-SIM setups, which were not equipped with an appropriate excitation laser and wavelength detection range. Still, apart from the vortices (which were too small to be identified on the confocal microscope), we were able to at least determine the positions of actin stars and asters in the confocal microscope and relate it to the amount of S-L2 fluorescence. Supplementary Figure 6A shows such two-color confocal image of the basal membrane in several representative HeLa cells showing only stress fibers and no noticeable actin patterns (e.g. white arrow left panel). As expected, S-L2 localized predominately to the membrane and fluorescence emission was observed along the membrane (e.g. white arrow in middle panel). To quantify the degree of overlap in space between F-actin and S-L2, we calculated the Squared-overlap-coefficient r^2 from the merged dual-color images. The Squared-overlap-coefficient is 1 for a high degree of overlap and smaller than 1 for reduced degree of overlap. In the case of the HeLa cells showing only stress fibers and no noticeable actin patterns, r^2 equaled 0.66 ± 0.05 (standard error of the mean, s.e.m.), indicating no strong localization of more ordered membrane environments towards the stress fibers. In the HeLa cells displaying stars (Supplementary Figure 6B and 7), r^2 was not significantly different ($p = 0.9$ student t-test, $r^2 = 0.61 \pm 0.05$ s.e.m.) within the regions of the stars (r^2 was computed in the region with stars being present, e.g. within the squared white box), highlighting as well no strong localization of more ordered membrane environments towards actin stars. Unexpectedly, the membrane order was significantly increased in regions where asters occurred after 12h incubation (Supplementary Figure 6C, $p < 0.01$ (student t-test) compared to the condition where no patterns or stars were present, $r^2 = 0.97 \pm 0.03$ (s.e.m.), Supplementary Figure 7), highlighting a high degree of overlap (white arrow in left and middle panels). This prompted us to also examine in the membrane environment in sparser aster regions (Supplementary Figure 6D). Consistently with the observations at larger fields of actin asters, individual asters surrounded by randomly organized F-actin displayed a significant increase in the membrane order compared to control conditions ($r^2 = 0.91 \pm 0.06$ s.e.m., $p < 0.01$ student t-test). These observations indicated that microscopic patterns in the form of asters indeed altered the architecture of the immediate environment around the asters, specifically increasing molecular lipid order.

Supplementary Note 7

Relations of actin patterns to podosomes. The actin patterns observed in our experiments seem structurally similar to actin podosomes, previously determined from SEM images [13]. Podosomes are also actin-rich, round structures at the vicinity of the basal plasma membrane. They are 0.5–2μm in diameter and play an important role in cellular motility and invasion, and are typically found in cells that specialize in these features. Yet, several characteristics reveal that the structures observed in our experiments were not podosomes. 1) The appearance of podosomes is not common in HeLa cells (which has been another reason for the choice of cell type in our investigations). However, podosome formation can be induced in HeLa cells by over-expression of members of the Src family kinases [14]. 2) In contrast to our SEM images of the cellular apical membrane, podosomes usually only appear at the cellular basal plane. 3) In contrast to the star-like actin features, podosomes do not undergo transitions or changes in their size following 30 min treatment by the Arp2/3-complex-inhibitor CK666. Rather, podosomes have been shown to completely disappear in THP1 monocytes after CK666 treatment [15]. 4). Myosin-II localizes to the arms AND core of podosomes [16], which strongly contrasts our observations. 5) Vinculin, a cytoskeletal protein that strongly localizes to podosomes [17], has not been found to coincide with the star-like structures of our recordings (Supplementary Figure 8).

Supplementary Discussions

At early times during cell adhesion, cells self-organize the cortical network into vortices that actively nucleate actin filaments from their periphery. Theoretical considerations of active gel theory predict the formation of vortices when the actin network undergoes a mechanical instability induced by a negative bending elastic modulus [18]. Such negative bending can be caused by cortical tension and frictional forces arising from the adhesion process of the basal cortex upon formation of focal adhesions during attachment to the substrate [19]. Theory also predicts that star-like structures are expected to form once mechanical stress leads to an effective positive bending modulus [18]. It is likely that the actin cortex of the HeLa cells undergoes a multitude of cortical actin rearrangements due to the changes in shape in response to the adhesion processes, which – as indicated here - may lead to the formation of large actin stars and subsequently into actin asters. How exactly positive and negative bending modes are achieved within the cortical network remains unclear.

Formation of vortices was also observed for microtubule in assays *in vitro* comprising microtubles and kinesin motor proteins [20]. Depending on the concentration of kinesin motor proteins, the isotropic microtubles network organized into different patterns such as rotating spirals, stars, and asters. Employing such self-organization provides adhering cells with fast and effective mechanisms to generate new structures. Rotating actin-rich vortices in particular efficiently polymerize new interconnected actin throughout all orientations within the two-dimensional vicinity of the basal membrane. Notably, the vortex morphology as observed in our experiments suggested no crosslinking to the peripheral cortical actin network (since no actin was visible in the vortex surroundings), which urges the question how the rotation of the vortices are physically achieved. eTIRF-SIM measurements demonstrated that myosin-II appeared to play no role in the nucleation and maintenance of self-organized actin vortices. Nevertheless, myosin-II may still contribute in addition to other known actin crosslinkers such as fascin and alpha-actinin to the generation of the mechanical intrinsic stress that is required for the initiation of the vortex formation. The actin-nucleation activity of the Arp2/3 complex likely is propulsive of actin vortex mobility, which, in the case that the vortex centers are linked to some other structure such as the membrane, leads to the rotation of the vortices. Eventually, the peripheral actin strands of the vortices are crosslinked to the surrounding F-actin and the vortices will transform into asteriks-like features such as stars, as highlighted by our experiments. In contrast to our observations, it in principle seems more feasible that vortices transform first into asters and then into stars, since the size of vortices and asters is comparable (and they might therefore easily transfer into each other) while stars are 5-6 fold larger.

After transition of vortices into stars and in the cause of full cell adherence to the substrate, the most dominant change was the transition from stars into asters. Specifically, the stars transformed into actin asters by dividing their actin cores and peripheral strands. The reverse transformation from asters to stars was predicted by Monte Carlo simulations [3] and *in vitro* experiments [4]. These experiments expected a strong influence on actin self-organization from enhanced myosin motor concentration and activity [21], which we could not confirm as a dominant process in the nucleation and maintenance of actin patterns in the cells. We could at one occasion also image multiple small asters transforming into one actin star by fusing their actin cores and strands. Still, this star was relatively small in size compared to other stars presented in this work, suggesting that star formation might require a series of fusion steps of multiple actin asters. Hence, great care has to be taken when directly transferring results from *in silico* and *in vitro*

experiments to the cellular conditions, and future *in silico* and *in vitro* experimental conditions have to be carefully designed to match those. For example, the cortex-membrane interface in the three-dimensional cellular environment is more complex than it has been modeled *in silico* and *in vitro* so far, involving dynamic interactions of cytoplasmic actin networks and organelles, ultimately highlighting the necessity of more complete models, considering for example the mechanical coupling to the membrane, the substrate, or cytoplasmic actin [9,22].

From a mechanical point of view, the morphology and stiffness of the substrate may also affect self-organized pattern organization during cell adherence. While we employed glass substrates without additional biological factors, similarly to the well-characterized experiments in vitro, soft-matter surfaces, as it would be provided by hydrogels functionalized with specific protein ligands such as integrins, may allow to study the effects of mechano-sensitivity and -transduction on self-organized cortex assembly. Intriguingly, we did not detect any obvious structural differences of the actin patterns in fixed and living cells. Even though the cells were exposed to biological and mechanically stressful treatments during cell fixation, self-organized pattern organization was reliably be observed throughout the cell cortex including the apical and basal cell planes. This may suggest that cellular self-organization is to some degree robust towards environmental changes.

Supplementary References

1 Verkhovsky, A. B., Svitkina, T. M. & Borisy, G. G. Polarity sorting of actin filaments in cytochalasin-treated fibroblasts. *J Cell Sci* **110**, 1693-1704 (1997).

2 Vignjevic, D. *et al.* Formation of filopodia-like bundles in vitro from a dendritic network. *J Cell Biol* **160**, 951-962, doi:10.1083/jcb.200208059 (2003).

3 Haviv, L. *et al.* Reconstitution of the transition from lamellipodium to filopodium in a membrane-free system. *Proc Natl Acad Sci U S A* **103**, 4906-4911, doi:10.1073/pnas.0508269103 (2006).

4 Ideses, Y., Brill-Karniely, Y., Haviv, L., Ben-Shaul, A. & Bernheim-Groswasser, A. Arp2/3 branched actin network mediates filopodia-like bundles formation in vitro. *PLoS One* **3**, e3297, doi:10.1371/journal.pone.0003297 (2008).

5 Soares e Silva, M. *et al.* Active multistage coarsening of actin networks driven by myosin motors. *Proc Natl Acad Sci U S A* **108**, 9408-9413, doi:10.1073/pnas.1016616108 (2011).

6 Riedl, J. *et al.* Lifeact: a versatile marker to visualize F-actin. *Nat Methods* **5**, 605-607, doi:10.1038/nmeth.1220 (2008).

7 Chiu, C. L., Digman, M. A. & Gratton, E. Measuring actin flow in 3D cell protrusions. *Biophys J* **105**, 1746-1755, doi:10.1016/j.bpj.2013.07.057 (2013).

8 Yamashiro, S. *et al.* New single-molecule speckle microscopy reveals modification of the retrograde actin flow by focal adhesions at nanometer scales. *Mol Biol Cell* **25**, 1010-1024, doi:10.1091/mbc.E13-03-0162 (2014).

9 Fritzsche, M. & Charras, G. T. Dissecting protein reaction dynamics in living cells by fluorescence recovery after photobleaching. *Nat Protoc* **10**, 660-680 (2015).

10 Gowrishankar, K. *et al.* Active remodeling of cortical actin regulates spatiotemporal organization of cell surface molecules. *Cell* **149**, 1353-1367 (2012).

11 Lim, C. S. *et al.* A two-photon turn-on probe for lipid rafts with minimum internalization. *Chembiochem* **12**, 392-395, doi:10.1002/cbic.201000609 (2011).

12 Sezgin, E., Sadowski, T. & Simons, K. Measuring lipid packing of model and cellular membranes with environment sensitive probes. *Langmuir* **30**, 8160-8166, doi:10.1021/la501226v (2014).

13 Luxenburg, C. *et al.* The architecture of the adhesive apparatus of cultured osteoclasts: from podosome formation to sealing zone assembly. *PLoS One* **2**, e179, doi:10.1371/journal.pone.0000179 (2007).

14 Tu, C. *et al.* Lysosomal cathepsin B participates in the podosome-mediated extracellular matrix degradation and invasion via secreted lysosomes in v-Src fibroblasts. *Cancer Res* **68**, 9147–9156 (2008).

15 Nolen, B. J. *et al.* Characterization of two classes of small molecule inhibitors of Arp2/3 complex. *Nature* **460**, 1031-1034 (2009).

16 van den Dries, K. *et al.* Interplay between myosin IIA-mediated contractility and actin network integrity orchestrates podosome composition and oscillations. *Nat Commun* **4**, 1412, doi:10.1038/ncomms2402 (2013).

17 van den Dries, K. *et al.* Dual-color superresolution microscopy reveals nanoscale organization of mechanosensory podosomes. *Mol Biol Cell* **24**, 2112-2123, doi:10.1091/mbc.E12-12-0856 (2013).

18 Kruse, K., Joanny, J. F., Julicher, F., Prost, J. & Sekimoto, K. Asters, vortices, and rotating spirals in active gels of polar filaments. *Phys Rev Lett* **92**, 078101, doi:10.1103/PhysRevLett.92.078101 (2004).

19 Shemesh, T., Bershadsky, A. D. & Kozlov, M. M. Physical model for self-organization of actin cytoskeleton and adhesion complexes at the cell front. *Biophys J* **102**, 1746-1756, doi:10.1016/j.bpj.2012.03.006 (2012).

20 Nedelec, F. J., Surrey, T., Maggs, A. C. & Leibler, S. Self-organization of microtubules and motors. *Nature* **389**, 305-308, doi:10.1038/38532 (1997).

21 Backouche, F., Haviv, L., Groswasser, D. & Bernheim-Groswasser, A. Active gels: dynamics of patterning and self-organization. *Phys Biol* **3**, 264-273, doi:10.1088/1478-3975/3/4/004 (2006).

22 Fritzsche, M., Thorogate, R. & Charras, G. Quantitative analysis of ezrin turnover dynamics in the actin cortex. *Biophys J* **106**, 343-353, doi:10.1016/j.bpj.2013.11.4499 (2014).

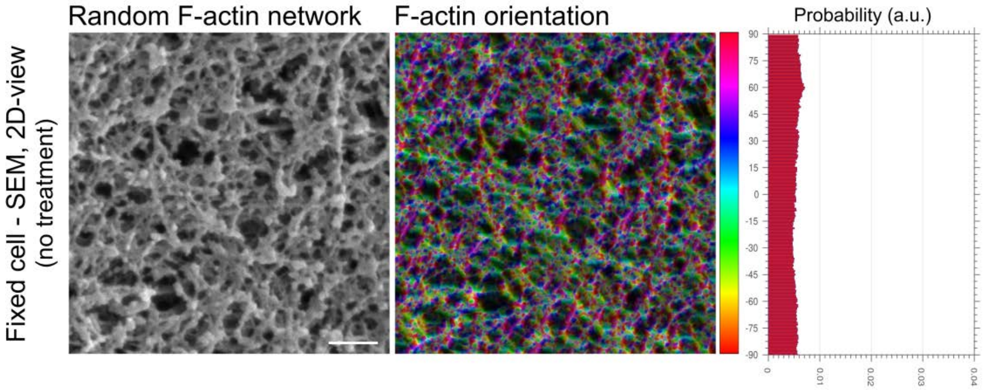

Supplementary Figure 1. Exemplification of the orientation analysis of the basis of a randomly organized F-actin network from SEM images. The frequency distribution of F-actin orientation is uniformly distributed. Scale bar 100 nm.

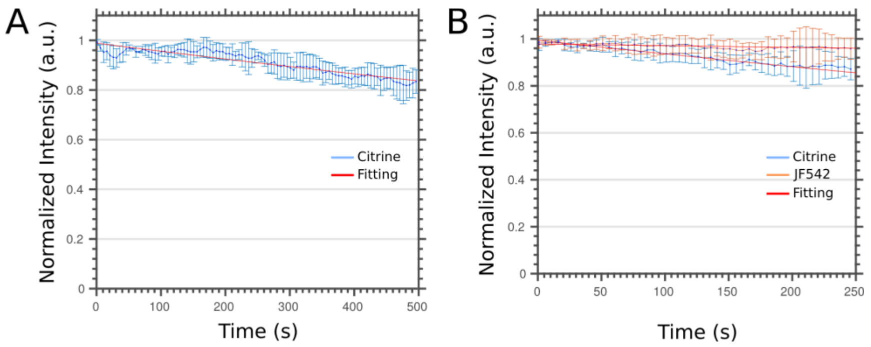

Supplementary Figure 2. Laser-induced photo-bleaching in the time-lapse eTIRF-SIM experiments. Relative loss in detected total fluorescence of a TIRF-SIM image over time. (**A,B**) The fluorescence loss curves for citrine (blue) and JF542 (orange) were fitted with a single exponential red line) and yielded a characteristic bleaching time-scale of 300 s in the case of citrine and 1000 s in the case of JF542. Error bars are standard deviations of the mean from 20 independent measurements.

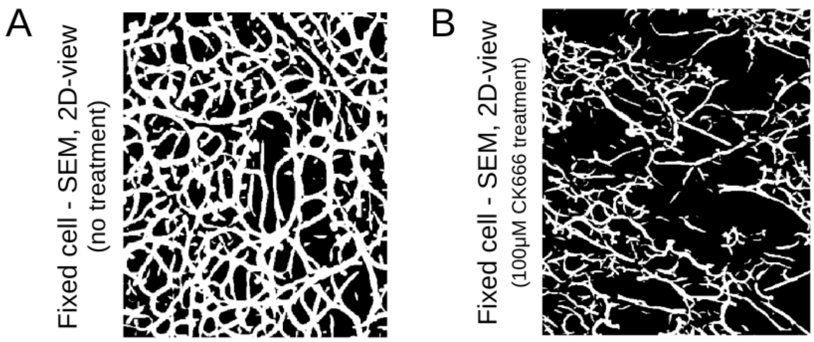

Supplementary Figure 3. Exemplification of the basis of the actin meshwork analysis from SEM images of fixed HeLa cells (Figure 3G). The average sizes of the cortical actin meshes were determined through a multi-stage image-processing protocol (see section ‘F-actin pore-size’). This analysis produced segmented (binarized) images of actin networks without treatment (**A**) and after treatment with CK666 (**B**), which were directly used for determination of the pore-size.

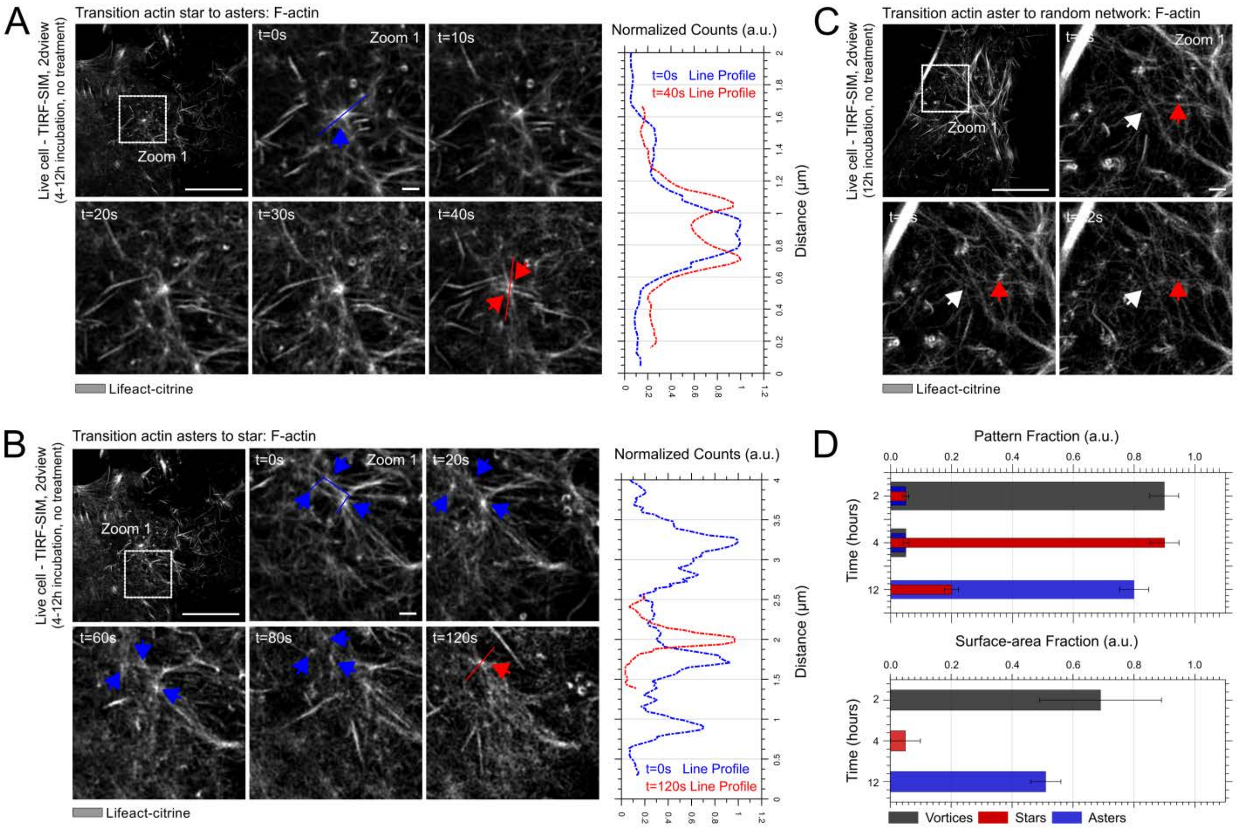

Supplementary Figure 4. eTIRF-SIM recordings of direct transition events between actin asters and stars at the basal membrane of living HeLa cells in-between 4-12 h incubation time. (**A-C**) Representative images of actin (Lifeact-citrine, grey scale), overviews (upper left,), and zoom-ins (into white boxes) at different time points of the image series (label t in the upper left corner, scale bar), and (right panels in A,B) intensity line profiles along red lines of images taken at t = 0 (blue) and 40 s (red), exemplifying (blue/white and red arrows in images) events of transitions between actin stars to asters (A), asters to stars (B) and disassembly of asters (C). (**D**) Fractions of totally identified patterns (upper panel) and fractions of total surface area taken (lower panel) of vortex (black), stars (red) and asters (blue) determined from eTIRF-SIM images taken at different incubation time points as labeled. Error bars are standard deviations of the mean from 120 cells of at least 3 independent experiments. Scale bars 5 µm (overviews) and 1 µm (zoom-ins).

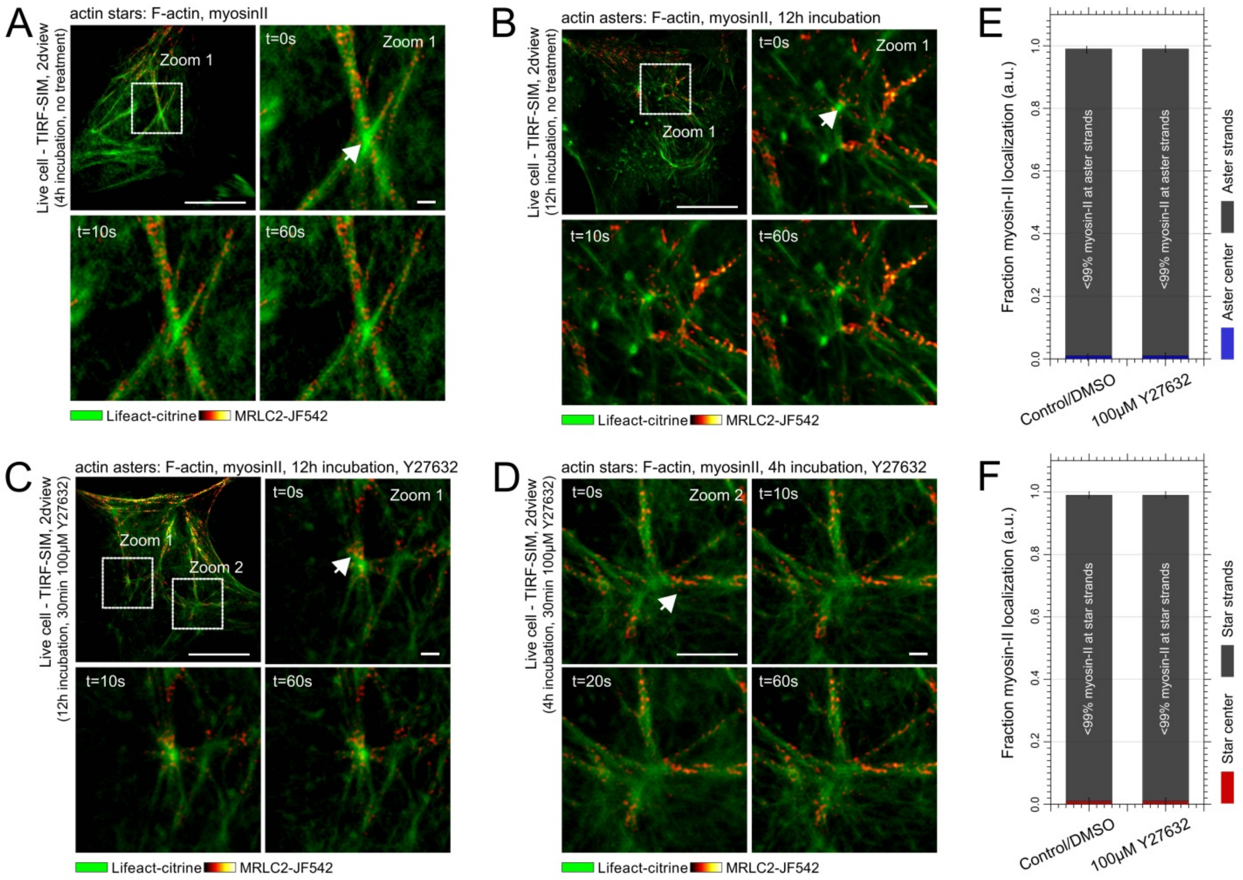

Supplementary Figure 5. eTIRF-SIM recordings of the dynamics of actin (Lifeact-citrine, green) and myosin-II (MRLC2-JF542, red to yellow) at the basal membrane of living HeLa cells. (**A-D**) Actin and myosin II without treatment after short (A, 4-5 h incubation, actin stars) and long (B, 12 h incubation, actin asters), depicting that myosin patches localized only to the peripheral F-actin of both stars and asters (not to their centers, white arrow), and (C, D) following treatment by the myosin inhibitor Y27632 (100 µM, 30 min, 12 h total incubation i.e. presence of asters), indicating blocking of myosin II activity (no mobility) but no influence on the maintenance of the asters: overviews (upper left in A-C), and zoom-ins into areas marked by white boxes at different time points of the image series (label t in the upper left corner). (**E, F**) Fraction of myosin-II localized to centers (black) and peripheral F-actin arms (blue and red) of asters (E) and stars (F) as taken from the images after short and long incubation, respectively, and without treatment, under DMSO control and after Y27632 treatment, as marked. Error bars are standard deviations of the mean from 120 cells of at least 3 independent experiments. Scale bars 5 µm (overviews) and 1 µm (zoom-ins).

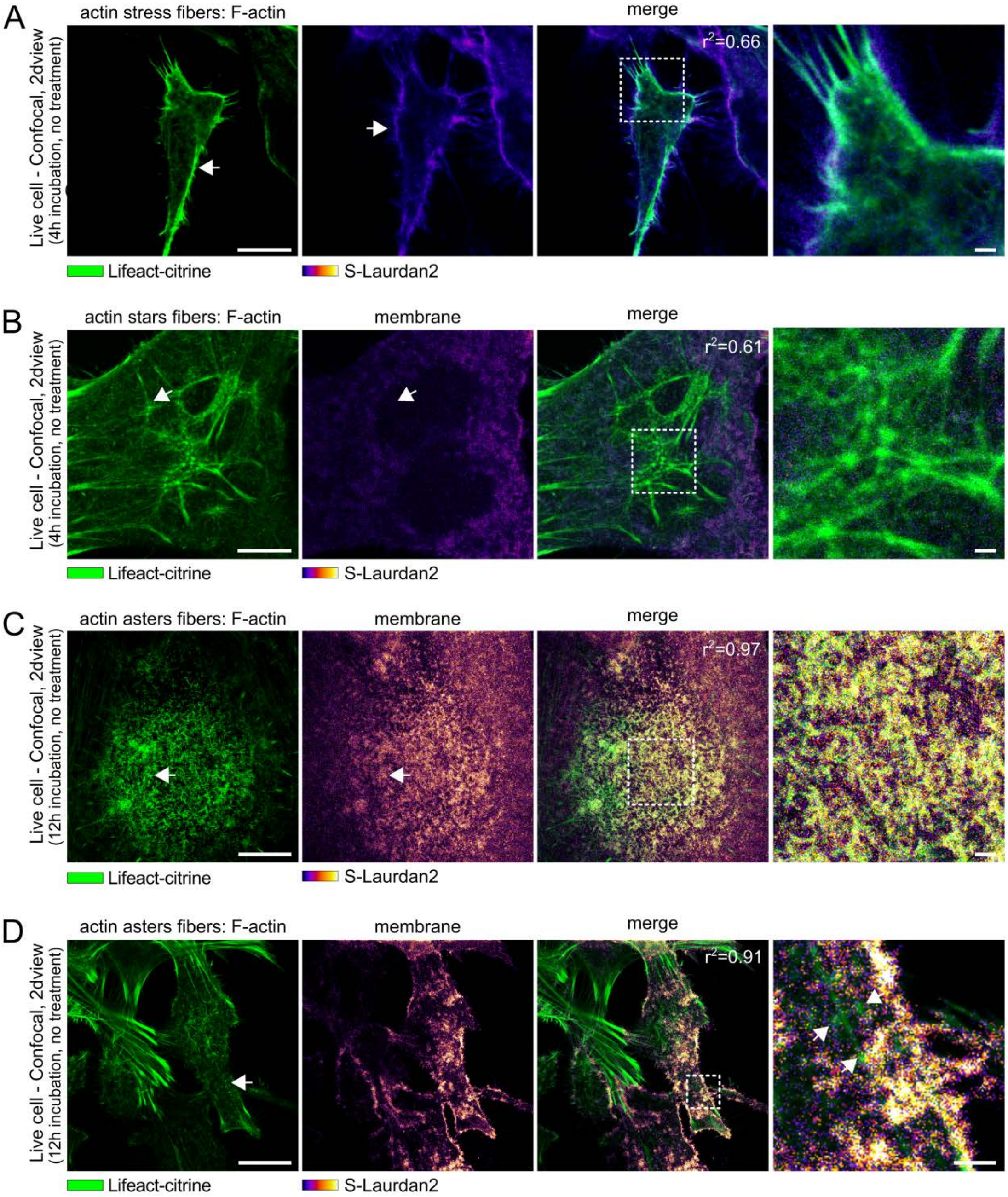

Supplementary Figure 6. Membrane fluidity measurements. Two-color confocal images of F-actin (green, left panels, Lifeact-citrine) and S-L2 membrane staining (black-to-yellow, second left panel) with merged overlay images (second right panels) and zoom-ins of the regions marked by the respective white-dotted boxes (right panels). Different conditions are shown: HeLa cells showing (**A**) only actin stress fibers, (**B**) actin stars and (**C,D**) actin asters. The different actin patterns are representatively highlighted by the white arrows. Strong S-L2 signal depicts areas with increased lipid membrane order (white arrows). Values of the Squared-overlap-coefficient r^2 are given as calculated from the merged dual-color images, with large values depicting a strong overlap between the respective actin patterns and ordered environments (compare Supplementary Figure 7). Scale bars 10 μm (overviews) and 1 μm (zoom-ins).

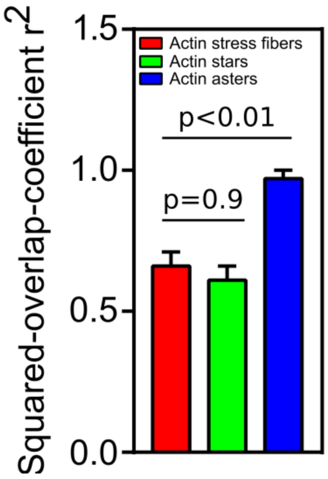

Supplementary Figure 7. Squared-overlap-coefficient r^2 of membrane fluidity measurements. Values of r^2 were measured around the actin patterns (stress fibers, stars and asters) for the merged image data of F-actin and the lipid order sensitive dye S-L2 in the basal membrane of live HeLa cells (see Supplementary Figure 6); the closer the value is to 1 the more the actin patterns affect membrane architecture by increasing lipid order/packing. Consequently, the influence from actin-rich stress fibers and actin stars was similarly moderate ($p = 0.9$ student t-test; $r^2 = 0.66 \pm 0.05$ s.e.m. and $r^2 = 0.61 \pm 0.05$ s.e.m.), while actin asters ($r^2 = 0.97 \pm 0.03$ s.e.m.) generate significantly different ($p < 0.01$ student t-test) highly ordered membrane environments. Error bars are standard deviations of the mean from 60 different cell images of at least 3 independent experiments.

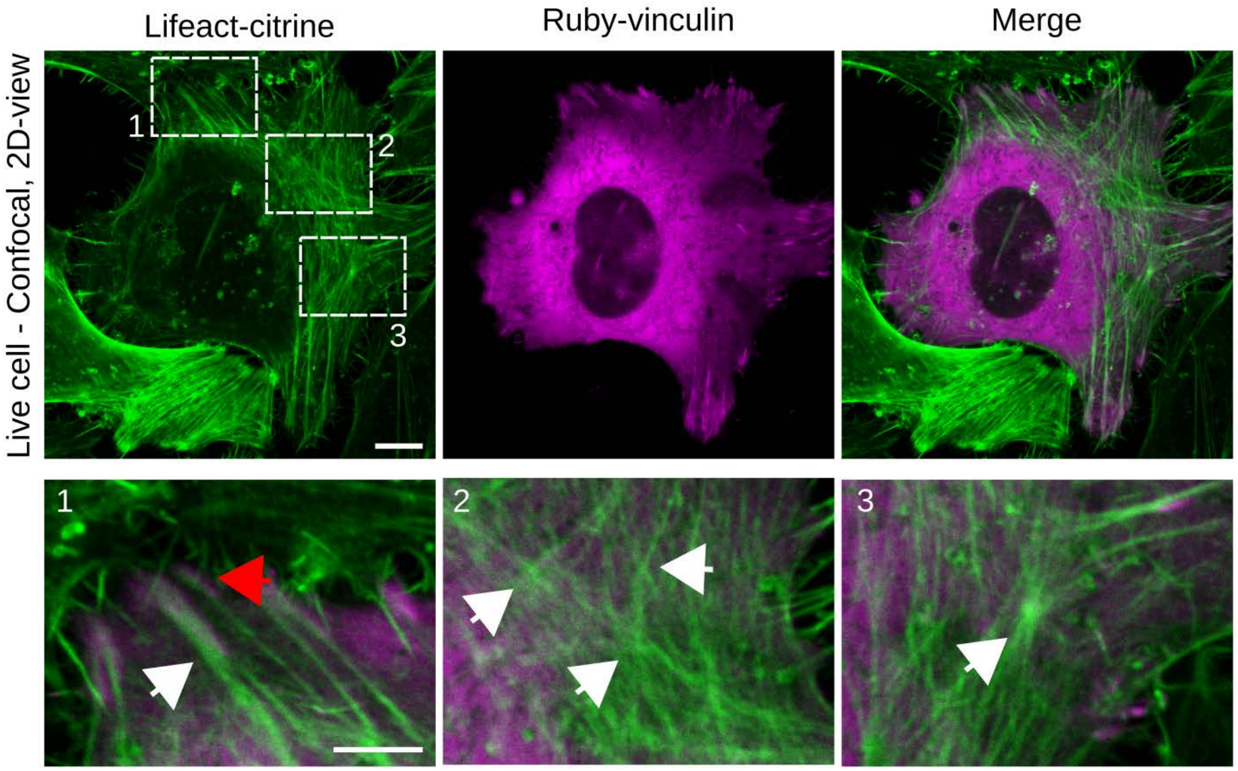

Supplementary Figure 8. Testing for podosomes. Confocal images of actin (left, Lifeact-citrine, green) and vinculin (middle, Ruby-vinculin, purple), and merged image (right): overview (upper panels) and zoom-ins 1-3 into white boxes (lower panels). Vinculin localized to the focal adhesion points of HeLa cells (white arrow zoom-in 1) but not to actin filaments (red arrow). Additionally, vinculin did not localize to the center of actin asters or stars (white arrows zoom-ins 2 and 3), as it would be expected for podosomes. Scale bars 10 μm (overviews) and 5 μm (zoom-ins).

Experiment	δ (μm)	ξ (nm)	p-value	N_δ	N_ξ
No treatment	5 ± 1.2	23 ± 20	< 0.01	58	2950
100μM CK666	2.5 ± 1	42 ± 52	< 0.01	98	3560

Supplementary Table 1: Summary of median aster diameter δ and median cortical actin pore-size ξ with no treatment and with 30min treatment by 100μM CK666. p-values compare the two conditions. N_δ is the number of measurements of the aster diameter and N_ξ of the actin pore-sizes.
